# A roadmap of cell-type specific gene expression during sequential stages of the arbuscular mycorrhiza symbiosis

**DOI:** 10.1186/1471-2164-14-306

**Published:** 2013-05-07

**Authors:** Claudia Hogekamp, Helge Küster

**Affiliations:** 1 Institut für Pflanzengenetik, Abteilung IV - Pflanzengenomforschung, Leibniz Universität Hannover, Herrenhäuser Str. 2, Hannover, 30419, Germany

**Keywords:** Arbuscular mycorrhizal symbiosis, Cellular expression profiling, Laser-microdissection, *Medicago* GeneChips, Sequential reprogramming, Transcriptional regulators

## Abstract

**Background:**

About 80% of today’s land plants are able to establish an arbuscular mycorrhizal (AM) symbiosis with *Glomeromycota* fungi to improve their access to nutrients and water in the soil. On the molecular level, the development of AM symbioses is only partly understood, due to the asynchronous development of the microsymbionts in the host roots. Although many genes specifically activated during fungal colonization have been identified, genome-wide information on the exact place and time point of their activation remains limited.

**Results:**

In this study, we relied on a combination of laser-microdissection and the use of *Medicago* GeneChips to perform a genome-wide analysis of transcription patterns in defined cell-types of *Medicago truncatula* roots mycorrhized with *Glomus intraradices*. To cover major stages of AM development, we harvested cells at 5-6 and at 21 days post inoculation (dpi). Early developmental stages of the AM symbiosis were analysed by monitoring gene expression in appressorial and non-appressorial areas from roots harbouring infection units at 5-6 dpi. Here, the use of laser-microdissection for the first time enabled the targeted harvest of those sites, where fungal hyphae first penetrate the root. Circumventing contamination with developing arbuscules, we were able to specifically detect gene expression related to early infection events. To cover the late stages of AM formation, we studied arbusculated cells, cortical cells colonized by intraradical hyphae, and epidermal cells from mature mycorrhizal roots at 21 dpi. Taken together, the cell-specific expression patterns of 18014 genes were revealed, including 1392 genes whose transcription was influenced by mycorrhizal colonization at different stages, namely the pre-contact phase, the infection of roots via fungal appressoria, the subsequent colonization of the cortex by fungal hyphae, and finally the formation of arbuscules. Our cellular expression patterns identified distinct groups of AM-activated genes governing the sequential reprogramming of host roots towards an accommodation of microsymbionts, including 42 AM-activated transcription factor genes.

**Conclusions:**

Our genome-wide analysis provides novel information on the cell-specific activity of AM-activated genes during both early and late stages of AM development, together revealing the road map of fine-tuned adjustments of transcript accumulation within root tissues during AM fungal colonization.

## Background

The arbuscular mycorrhiza (AM) symbiosis represents one of the most ancient and widespread symbioses known. Around 80% of all land plants establish this interaction with fungi of the phylum *Glomeromycota*[1]. Fossil records date back over 450 million years and coincide with the appearance of the first land plants, indicating that the symbiosis may have been an important factor during the colonization of terrestrial ecosystems by plant species [2]. In the course of the symbiosis, roots are colonized by fungal hyphae that ultimately form intracellular tree-like structures termed arbuscules in the inner-cortical cells, facilitating nutrient exchange between the two partners [3]. While the microsymbiont is supplied with photoassimilates from the host [4], the plant benefits from the wide-stretched network of extraradical fungal hyphae in the soil, providing access to phosphate, nitrate, minerals, and water [5,6]. The establishment of such an intimate interaction, allowing the fungus to grow intracellularly in the host cells, demands for its recognition as a symbiotic partner by the plant and a tight regulation of processes leading to the accommodation of the beneficial fungus. On the molecular level, this process is only partly understood and the exact place and time point of activation, as well as the precise function of most genes known to be upregulated during fungal colonization of the host plant remain elusive. To some extent, this is due to the way mycorrhizal fungi proliferate in the host roots. During the symbiotic interaction, different developmental stages can be distinguished, ranging from the pre-contact phase, where both symbiotic partners communicate via diffusible signal molecules [7-10] followed by appressorium formation on the root epidermis and initial infection to the spread of fungal hyphae in the outer and finally arbuscule formation in the inner cortex [3]. While all of these stages are present in the infected roots within hours after the first contact between both partners, the further progress of the symbiosis is characterized by permanent arbuscule build-up and break-down [11]. At the same time, colonization of new root areas occurs in parallel via intraradical hyphae, as well as growth of extraradical hyphae and the formation of new appressoria. This asynchronous development leads to the concomitant presence of all developmental AM stages when root colonization reaches sufficient levels for molecular analyses, making it extremely difficult to relate gene activity to distinct AM stages.

In addition to high-throughput transcriptome studies based on whole mycorrhizal roots dominated by later stages of the symbiotic interaction, which identified sets of AM-induced genes [12-17], several attempts were undertaken in recent years to enrich mycorrhizal roots for early symbiotic stages. Here, suppressive subtractive hybridization (SSH)-based expression profiling, EST sequencing, and proteome analyses of *M. truncatula* roots colonized with AM fungi for up to ten days resulted in the identification of a few plant genes potentially related to appressorium formation [18-21].

One of the first reactions of root cells to the presence of mycorrhizal fungi is the occurrence of a characteristic calcium-spiking in the cytoplasm and the nucleus [22], which is decoded by the calcium-dependent protein kinase DMI3 [23]. The usage of *dmi3*-mutant plants allowed a first classification of AM-induced genes with regard to their activation upstream or downstream of the calcium-spiking initiated upon recognition of the microsymbiont [19-21]. Another indication of a forthcoming infection is the formation of an intracellular prepenetration apparatus (PPA) consisting of a membrane tunnel surrounded by cytoskeletal components, which guides the invading hyphae through the epidermal cells [24,25]. Siciliano *et al*. [26] used PPA formation as a marker for an initiating infection and selected those areas where PPAs were visible for the construction of an SSH library, thereby circumventing dilution effects which hampered earlier investigations. Nevertheless, an important draw-back was that the presence of young arbuscules could not be excluded completely, and the genes identified might therefore be related to later AM stages as well [27].

A commingling of different stages can only be avoided by analyses restricted to defined cell-types obtained via laser-microdissection. First applications of this method revealed differential expression patterns between arbusculated and the surrounding cortical cells for subsets of genes identified via global transcriptome analyses of whole AM roots from tomato [28,29] and *Lotus japonicus*[30] In *M. truncatula*, such studies initially revealed the specific expression of 27 genes in arbusculated cells [31] as well as the differential expression of 62 genes in arbusculated vs. the surrounding cortical cells [32] isolated from wax-embedded tissue sections. Relying on cryo-sections, Gaude *et al*. [33] recently combined laser-microdissection with a genome-wide transcriptome analysis to compare gene expression in arbusculated cells and cortical cells from mycorrhizal roots to control cells from uninfected roots.

We here report the use of laser-microdissection of wax-embedded root tissues for a comprehensive inventory of gene expression during key developmental stages of the AM symbiosis, using roots of *M. truncatula* colonized with *G. intraradices* (recently renamed *Rhizophagus irregularis*[34]). Genome-wide AM-related transcription was monitored for five different cell-types comprising not only arbusculated and surrounding cortical cells colonized by fungal hyphae, but for the first time also epidermal cells from mature mycorrhizal roots as well as appressorial and non-appressorial areas of roots containing first infection units. From a total of 18014 genes whose expression was detected in any cell-type, a subset of 1392 genes, including 42 that encode transcriptional regulators, displayed a cellular expression pattern influenced by mycorrhizal colonization, revealing their activation at specific stages of the symbiotic interaction. Distinct subsets of genes were found activated during the pre-contact phase, the initial fungal infection, the spread of intraradical hyphae, and arbuscule formation. Finally, genes downregulated during infection or arbuscule formation as well as a large group of genes which displayed a shift of expression during mycorrhization were identified. Taken together, these transcription patterns revealed a road map of fine-tuned adjustments of transcript accumulation within root tissues during the process of AM fungal colonization.

## Results and discussion

### Obtaining transcript sequences representing five microdissected cell-types from AM roots

**Figure 1:**
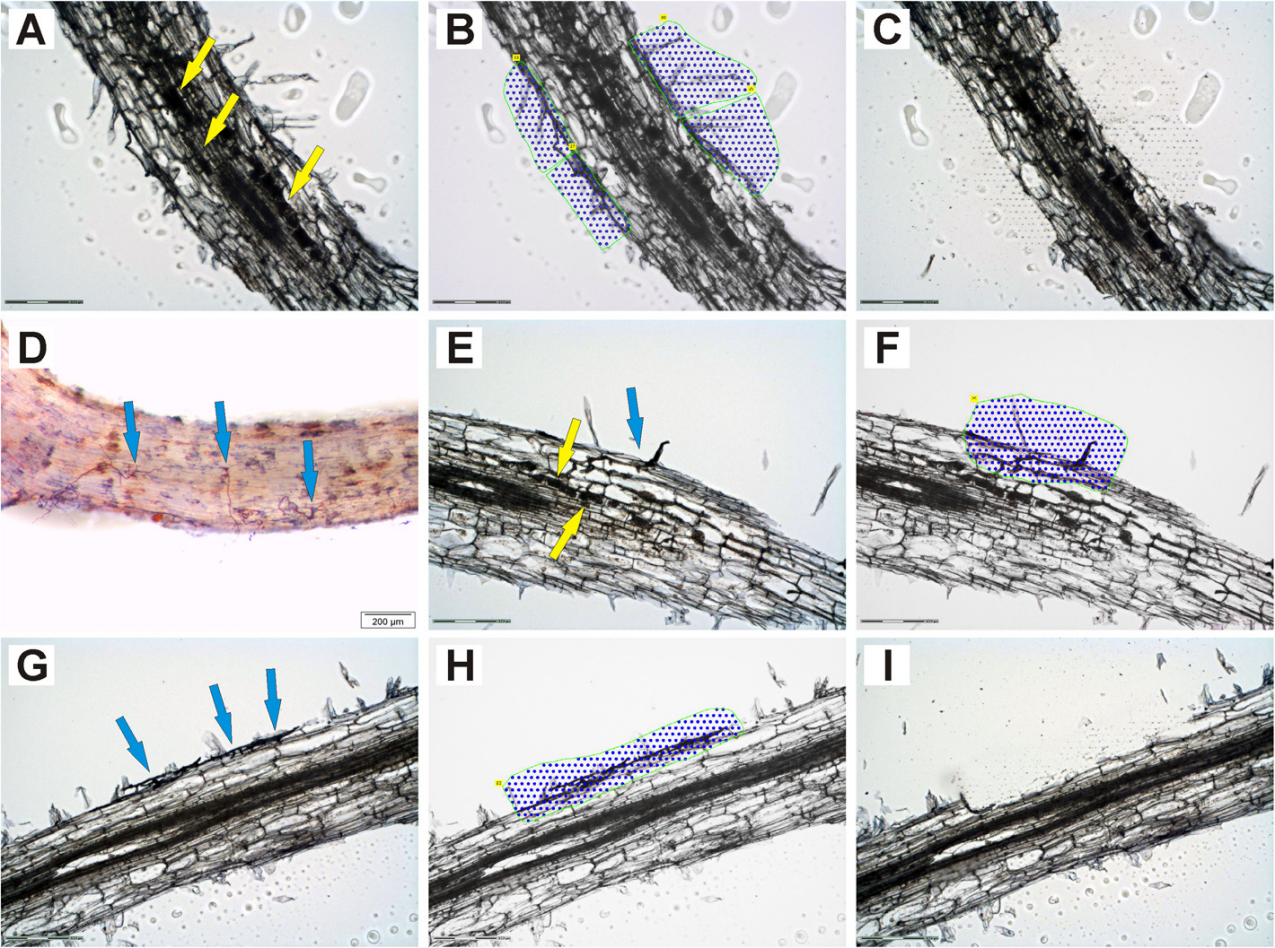
**Laser-microdissection of epidermal cells from AM roots and of root areas containing appressoria.** Collected areas are marked with blue dots framed in green. Arbuscules and appressoria are marked with yellow and blue arrows, respectively. Epidermal (EPI) samples (**A-C**) were collected from root areas containing mature mycorrhizal structures. The isolation of ARB and CMR cells from these samples has already been described in [32]. For the harvest of appressorial (APP) areas (**D-I**), roots were subjected to a short ink staining and screened for early infection units prior to embedding (**D**). During laser-microdissection, epidermal regions where fungal hyphae were penetrating the root and the cortical cells beneath were harvested for APP areas (**F, H**). In cases where arbuscules had already developed in the inner cortical cells (**E, F**), those cells were not included. Scale bars represent 200 μm for **D** and 150 μm for all other panels.

*M. truncatula* roots mycorrhized with *G. intraradices* (recently renamed *Rhizophagus irregularis*[34]) were used to obtain total RNA from pools of five specific cell-types via laser-microdissection. Cortical cells from mycorrhizal roots containing fungal hyphae (CMR) and cortical cells containing arbuscules (ARB) were collected at 21 days post inoculation (dpi) as described previously [32]. In addition, epidermal cells from mycorrhizal roots (EPI) were collected, taking care to harvest these only from areas containing fungal structures (Figure 1A-C).

Via pre-staining with ink, we were now able to collect pools of root cells containing appressoria (APP). In this case, cells were harvested already at 5-6 dpi from areas where extraradical hyphae had attached to the surface (Figure 1D). Comparable pools with no obvious fungal colonization from the same root systems were used to harvest non-appressorial areas (NAP). In 12 μm thin sections of the wax-embedded roots, appressoria were still clearly visible (Figure 1E,G). During laser-microdissection, a small area was collected, comprising epidermal cells in contact with the penetrating fungal hyphae and the colonized outer cortical cells beneath (Figure 1F,H,I). Although roots were harvested at the earliest possible time point showing fungal structures on the root surface, infection units had in some cases already proceeded to arbuscule development (Figure 1E). These appressorial areas were also collected, but care was taken not to include inner cortical cells containing arbuscules (Figure 1F) to avoid contamination with transcripts from later AM stages. For NAP samples, we harvested corresponding non-colonized areas of epidermal and cortical cells.

Two-round amplified biotinylated aRNAs from three biological replicates of each cell-type were used for *Medicago* GeneChip [35] hybridizations. The complete datasets are included in Additional file 1 (ARB, CMR, EPI) and Additional file 2 (APP, NAP). Due to their different origins, the datasets referring to mature mycorrhizal roots (ARB, CMR, EPI) or early infection events (APP, NAP) were analyzed separately both for detectable expression in single cell-types and for expression differences between them.

### Expression of 18014 genes was detected in microdissected cell-types from AM roots

We first analyzed whether genes were expressed in the different cell-types at all. Since we already demonstrated that transcripts of the arbuscule-specific phosphate transporter gene *MtPt4*[36] can only be detected by real-time RT-PCR in ARB samples [32], we used the *MtPt4* expression level as a biological threshold. As expected, *MtPt4* showed high mean signal intensities in the ARB samples (10.77), while mean signal intensities of *MtPt4* were extremely low in CMR and EPI (2.58) as well as in APP and NAP samples (2.95) that did not contain arbuscules. Consequently, those genes showing a mean signal intensity above 2.58 in ARB, CMR, and EPI samples or 2.95 in APP and NAP samples were regarded as expressed in the respective cell-types, while those with a lower value were classified as non-expressed. The validity of this classification is underlined by the fact that 14 of the 25 genes we identified as ARB-specific via real time RT-PCR [32] were again only expressed in this cell-type in our GeneChip hybridizations, while four genes were strongly ARB-induced (log_2_FC between 6 and 8.5). This general congruency indicates that the use of an *MtPt4* expression threshold should lead to a reliable identification of ARB-expressed genes and a correct estimate of cellular gene transcription in AM roots in general.

**Figure 2:**
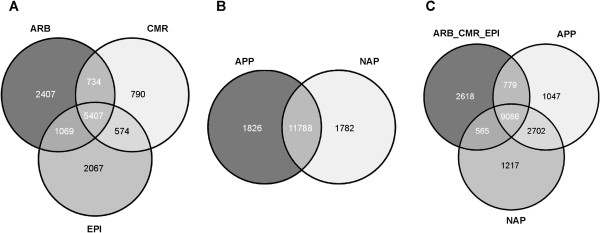
**Gene expression in five cell-types of AM roots. A**: Overview for ARB, CMR, and EPI cell-types. Genes were classified as expressed in a cell-type, if the corresponding mean signal intensity was larger than the threshold 2.58. **B**: Overview for APP and NAP cell-types. Genes were classified as expressed, if the corresponding mean signal intensity was larger than the threshold 2.95. Threshold values indicate the expression level of the arbuscule-specific *MtPt4* gene in the respective cell-types. **C**: Overlap of genes expressed in ARB_CMR_EPI, APP or NAP. Abbreviations: ARB, cortical cells containing arbuscules; CMR, cortical cells from mycorrhizal roots; EPI, epidermal cells from mycorrhizal roots; ARB_CMR_EPI, non-redundant sum of genes expressed in ARB, CMR, and EPI cell-types; APP, appressorial areas; NAP, non-appressorial areas.

Applying this threshold, we identified 13048 genes as expressed in either one, two or all three cell-types from mature mycorrhizal roots (Figure 2A, Additional file 3). As expected, the largest number of genes was expressed in all three cell-types (5407), while a considerable number was only expressed in ARB (2407), EPI (2067), or both cell-types (1069). Smaller but still considerable groups of genes were transcribed only in CMR (790), CMR and ARB (734), or CMR and EPI (574). In the two different cell-types from roots harbouring first infection units, 1826 genes were transcribed only in APP, 1782 only in NAP, and 11788 genes in both cell pools, resulting in a total number of 15396 expressed genes (Figure 2B, Additional file 4). As expected, a marked overlap between both datasets exists, since APP and NAP cells contained cortical and epidermal tissues (Figure 2C). Taken together, 18014 genes were classified as expressed in at least one of the five cell-types investigated (Figure 2C).

To obtain an estimate how many genes are transcribed in AM root tissues at all, a comparison to gene expression in whole roots colonized either with *G. intraradices* or *G. mossae*[32] was performed. Again, the expression level of *MtPt4* (3.38) in non-mycorrhizal roots under low phosphate supply was used as a threshold. This analysis resulted in a total of 31337 genes expressed in whole AM roots. Thus, we in total detected expression of appr. 50% of these in our five cell-type specific samples. Two facts probably account for the absence of transcripts from the remaining genes. First, RNA degradation, which cannot be avoided completely during the preparation of tissues for laser-microdissection, may lead to an overrepresentation of 3′ mRNA regions and/or a complete loss of mRNAs for less abundant transcripts, resulting in poor signal intensities in GeneChip hybridizations. Second, genes will be expressed in tissues or cell-types which were not included in the current analysis, e.g. vascular tissues or meristematic regions of main or lateral root tips. On the other hand, it is important to note that 1991 genes were detected as expressed in our cell-type specific samples, but not on the level of whole AM roots. In these cases, the isolation of specific cell-types probably led to the detection of low abundance transcripts which are otherwise lost due to dilution effects. This is supported by the observation that the portion of these genes was especially high for those expressed in epidermal cells, which can be expected to be particularly underrepresented in whole root tissues.

### Classification of AM-expressed genes according to their cell-type specific transcription pattern

**Figure 3:**
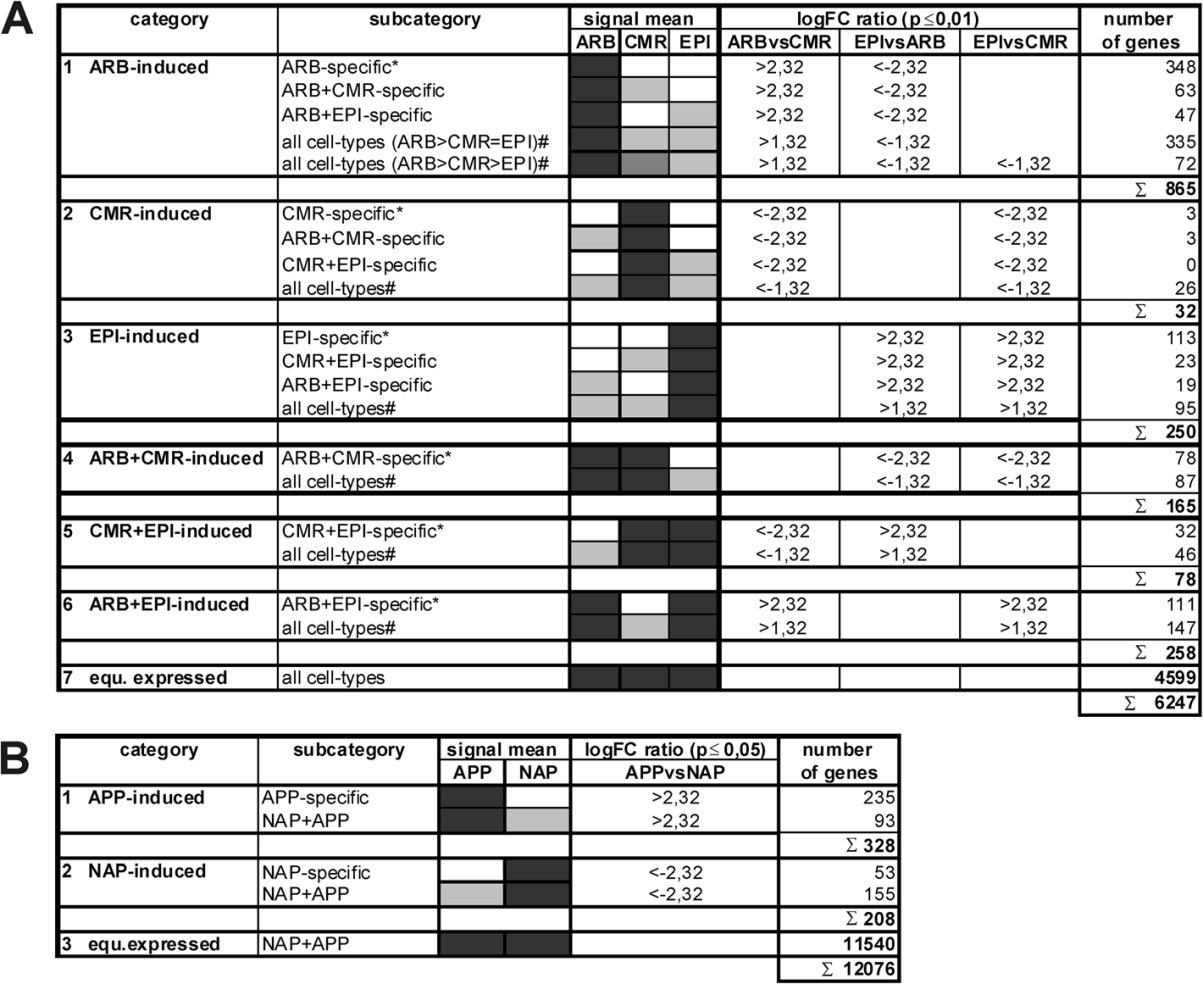
**Classification of genes according to their specific expression patterns in five cell-types of AM roots. A**: Genes expressed in ARB, CMR, and EPI were classified into seven categories. **B**: Genes expressed in APP and NAP were classified into three categories. While the 10 categories were defined on the basis of significant expression differences, all subcategories were differentiated based on detectable gene expression, using the *MtPt4*-thresholds as a reference. Grey-scaled boxes visualize different expression levels in the respective cell-types, ranging from white (no expression) to black (strong expression). Abbreviations: ARB, cortical cells containing arbuscules; CMR, cortical cells from mycorrhizal roots; EPI, epidermal cells from mycorrhized roots; APP, appressorial areas; NAP, non-appressorial areas; equ., equally; ind, induced; logFC, log2 fold-change; p, p-value. *To emphasize the importance of specific gene expression, genes were also considered as specific for a respective cell-type(s), if only one logFC ratio was above the threshold; # genes were also grouped into this subcategory if only one of the logFC ratios was significant; ∑: sum of genes in one category.

In a second step, we now classified genes according to significant expression differences between the cell-types investigated. For mature mycorrhizal stages, we applied a log_2_FC-threshold of 2.32 (5-fold expression ratio) at p≤0.01 for genes with expression above the *MtPt4* threshold in one or two cell-types, whereas for genes which were transcribed in all three cell-types, a lower log_2_FC-threshold of 1.32 (2.5-fold expression ratio) was used. Based on these conditions, we identified 1648 genes with differential expression in ARB, CMR, and EPI, while 4599 were equally expressed. All 6247 genes were grouped into seven categories (Figure 3A). Whereas the first three categories showed induction in a single cell-type (ARB, CMR, or EPI), the subsequent three categories contain genes induced in two cell-types in comparison to the third (ARB+CMR, CMR+EPI, or ARB+EPI). In each of these categories, genes were further subclassified based on their expression level, to account for cell-type specific expression as well (Figure 3A). Thus, in the first category of 865 ARB-ind genes, 348 in fact have to be classified not only as significantly ARB-induced but as ARB-specific, since they were not expressed above the *MtPt4* threshold in CMR and EPI (Figure 3A). In addition, 63 and 47 genes were only expressed in ARB+CMR and ARB+EPI, respectively. Finally, 407 of the ARB-induced genes were transcribed in all three cell-types, but displayed two different expression patterns. Whereas 335 of these were strongly induced in ARB with no significant difference between CMR and EPI (ARB>CMR=EPI), 72 displayed expression levels decreasing from ARB (ARB>CMR>EPI). Where possible, the other categories were subdivided accordingly (Figure 3A).

With respect to expression data from early infection events, we applied a log_2_FC-threshold of >2.32 (5-fold expression ratio) at p≤0.05 to identify significant expression differences between APP and NAP (Figure 3B). The lower p-value was applied, since these samples consisted of cortical and epidermal cells instead of one specific cell-type and we therefore expected higher variability. Whereas 328 genes were significantly induced in the APP, 208 genes were induced in the NAP cell-type. Together with the 11540 genes equally expressed in APP and NAP, 12076 genes were transcribed in areas prone to fungal infection.

### Cell-type specific expression of key AM marker genes

**Figure 4:**
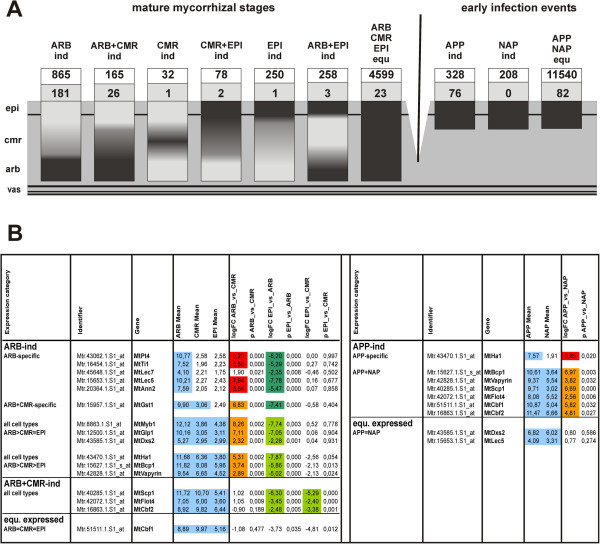
**Gene expression in mature mycorrhizal stages and early infection events including AM marker genes. A**: Visualization of gene expression patterns in AM roots for the seven categories referring to mature mycorrhizal stages and the three categories referring to early infection events, as defined in Figure 3. Overlaps between gene expression in mature mycorrhizal stages and early infection events is not shown in this diagram for reasons of simplicity. Numbers in white boxes represent the total number of genes in an expression category, numbers in grey boxes denote the overlap of this category with the 532 genes identified as “AM core set" due to a significant activation in whole roots by different AM fungi [32]. In total, cellular expression patterns were identified for 293 of all 532 genes from the AM core set. **B**: Detailed results for 16 AM marker genes, including mean signal intensities in the cell-types investigated, with signal intensities above the *MtPt4* expression threshold being marked in blue. Expression differences between the cell-types are indicated as follows: red/dark green: expression difference (logFC≥2.32; p≤0.01) for genes expressed above the *MtPt4* threshold in only one of the cell-types compared, orange/green: expression difference (logFC≥2.32; p≤0.01) for genes expressed above the *MtPt4* threshold in both cell-types compared. Abbreviations: ARB, cortical cells containing arbuscules; CMR, cortical cells from mycorrhizal roots; EPI, epidermal cells from mycorrhizal roots; APP, appressorial areas; NAP, non-appressorial areas; vas, vascular tissue; ind, induced; equ., equally expressed; logFC, log2 fold-change; p, p-value.

In a next step, we investigated whether we identified the correct cellular expression of AM-related genes in mature mycorrhizal roots and early infection events, based on the 10 categories defined in Figure 3. To this end, a comparison with the AM core set consisting of 532 genes significantly induced at least 2-fold both in roots colonized with *G. intraradices* and *G. mossae*, but not in roots treated with additional phosphate [32], was performed (Additional files 4 and 5). The results are shown in Figure 4, including a visualization of the derived expression patterns in AM roots (Figure 4A) and a table with detailed results for 16 AM marker genes (Figure 4B). Whereas a total of 237 genes from the AM core set were part of one of the seven categories from mature mycorrhizal stages, 158 were part of the three categories from early infection events. After eliminating overlaps between the two datasets, cellular expression patterns could be identified for 293 genes of the AM core set. Of the remaining 239 genes, 103 were expressed in at least one of the five investigated cell-types, giving first hints on their function as well, while 136 had not been transcribed in any cell-type, probably due to reasons discussed above.

Most genes of the AM core set [32], for which a cellular expression pattern was identified, belonged to the category of ARB-induced genes (181) (Figure 4A), confirming that transcripts from this cell-type dominate expression detected in whole root tissue. With regard to the subcategories, most of them were ARB-specific (87), including AM marker genes known to be specifically expressed or highly upregulated in this cell-type such as *MtPt4*[36], *MtTi1*[37], the two lectin genes *MtLec5* and *MtLec7*[14], and *MtAnn2*[38] (Figure 4B). The glutathione-S-transferase gene *MtGst1* was found to be ARB-induced, but also expressed in the surrounding cortical cells (Figure 4B), which is in line with the results of Wulf *et al*. [13]. The transcription factor gene *MtMyb1*[12], the *MtGlp1* gene encoding a germin-like protein [39], and *MtDxs2*[40] were detected as expressed in all cell-types with a strong induction in ARB in comparison to CMR and EPI, whereas the ATPase gene *MtHa1*[41], the *MtBcp1* gene encoding a blue copper protein [15] and *MtVapyrin*[42] were found in the subcategory of genes with an expression gradient from ARB to EPI (Figure 4B).

A second major part of the AM core set consists of genes induced in ARB+CMR alike (26) (Figure 4A), including the serine carboxypeptidase *MtScp1*, which was first identified and shown to be related to fungal spread in the cortex by Liu *et al*. [12], and the flotillin gene *MtFlot4* (Figure 4B) related to root nodule infection [43]. The two CAAT-box transcription factor genes *MtCbf1* and *MtCbf2*, recently shown to be active during all stages of mycorrhizal colonization [32], were expressed in all three cell-types (Figure 4B). While *MtCbf2* was significantly induced in ARB and CMR in relation to EPI, such activation was not significant for *MtCbf1*, which was therefore grouped into the category of equally expressed genes. This effect mirrors the slightly higher activity of *MtCbf1* in epidermal cells, which was already evident from histological studies [32]. Apart from *MtCbf1*, 22 genes of the AM core set were equally expressed in all three cell-types from mature mycorrhizal roots. The overlap to the remaining expression categories was very small, (Figure 4A, Additional file 5).

While no genes from the AM core set were NAP-induced, a higher overlap existed to genes which were either APP-induced (76) or equally expressed in APP and NAP (82) (Figure 4A). As expected from their activation by the first physical contact between plant and fungi [32], *MtCbf1* and *MtCbf2* were found to be APP-induced (Figure 4B). In addition, the AM marker genes, *MtHa1*, *MtBcp1*, *MtVapyrin*, *MtScp1*, and *MtFlot4* were already activated at this early stage, while *MtLec5* and *MtDxs2* were equally expressed in APP and NAP (Figure 4B).

### Appressorial cell pools are enriched for fungal genes

Since APP cell pools were harvested from regions of the root containing fungal material, we expected to find a substantial number of *G. intraradices* genes induced here. Therefore, a search for fungal probe sets was performed for all APP-induced genes by comparing the corresponding sequences from the *Medicago* Gene Expression Atlas (GEA) [35] to available *Glomus* sequences [44]. Blast hits with E≤0.001 were considered further, and if these had no match in *M. truncatula* sequences [45], they were classified as fungal genes. This resulted in a number of 202 fungal sequences among the APP-induced genes, which was roughly two third of all genes induced in this cell pool. Due to limited sequence similarities, most of these had been specifically “induced” only by *G. intraradices* and not by *G. mossae* on the level of whole roots, or were considerably less activated by *G. mossae*. Looking into genes from other categories, which showed a *G. intraradices* specific induction, we found another 10 fungal genes in addition to five fungal genes that had already been identified earlier [32], leading to a total number of 217 fungal genes (Additional file 6).

In Additional file 7, a functional classification of fungal genes is shown. Except of genes encoding proteins with unknown function (100), the three most prominent classes were translation (28), posttranslational modification of proteins (19), and amino acid transport and metabolism (11). Whereas the first two contain several fungal housekeeping genes, the third class represents an important part of the nutrient exchange network between AM fungi and plants, since nitrogen is transported in the fungal mycelium after incorporation into amino acids [46].

Apart from being APP-induced, most fungal genes showed either no cell-specific expression or were not transcribed in cell-types from mature AM roots (82 and 34 genes, respectively). Since APP cell pools contained considerable parts of extraradical hyphae, those genes might be preferentially expressed in the extraradical mycelium. The remaining genes (Additional file 6) were either ARB-induced (57), ARB+CMR-induced (29), or equally expressed in all three cell-types (15).

To get an impression how fungal material is distributed in these cell-types, we had a look at a housekeeping gene encoding the fungal translation elongation factor GiTefα (Mtr.4378.1.S1_at). As expected, *GiTefα* transcripts were specifically detected in APP and not in NAP (Additional file 6), while they displayed a gradient in mature mycorrhizal roots (ARB>CMR>EPI), indicating that fungal material is not equally distributed. This is plausible, since arbusculated cells contain highly ramificated intracellular hyphae, whereas in other cortical cells, hyphae mainly grow in the extracellular space, and epidermal cell pools will at most contain single hyphae growing on the surface. Due to this, fungal genes classified as ARB-induced, at least those with an expression gradient similar to *GiTefα*, have to be treated with caution, since they might indeed be equally expressed within fungal tissues, while the 29 fungal genes specifically transcribed in ARB probably show true differential expression.

### Genes constitutively expressed in root tissues

Although some exceptions exist, which are discussed below, most of the 4250 plant genes expressed at equal levels in ARB, CMR, and EPI, and in addition in APP and NAP (Additional file 8B), can be considered as constitutively transcribed in our experimental conditions, irrespective of fungal colonization. The housekeeping gene *MtTefα*[13], encoding the translation elongation factor α, indeed displayed an equal expression across all investigated cell-types in both datasets (Additional file 5).

Furthermore, in addition to genes transcribed in all cell-types, candidate genes with tissue-specific expression either in the root epidermis or the cortex can be found, which are nevertheless not influenced by the AM symbiosis. This is in particular the case for the 250 EPI-induced genes (Figure 3A), since comparisons to the *Medicago* Gene Expression Atlas [35] revealed that many of these were expressed in all other plant organs as well, suggesting that they contribute to a basic protein equipment of epidermal cells. Nearly no overlap was found to genes induced by fungal colonization on the level of whole roots (Additional file 8C) and a considerable part of EPI-induced genes displayed no clear expression pattern in APP and NAP samples (38 genes, Additional file 8B) or was not expressed in these cell-types at all (35 genes, Additional file 8B). The genes concerned were likely candidates for preferential activation in epidermal cells during later stages of the symbiosis, which was not identified on the level of whole roots due to dilution effects. Nevertheless, when gene function is regarded, this does not seem to be the case, since those genes mainly code for ribosomal proteins or parts of the respiratory chain (Additional file 4). The specific detection of the corresponding transcripts in epidermal cells from mature mycorrhizal roots may therefore be due to slight age differences of the plants used in the two approaches. Alternatively, a dilution of epidermal cells with cortical cells in APP and NAP samples may have led to a less efficient detection of transcripts from epidermal cells in those samples. Notably, nearly all of the genes concerned were EPI-specific, making a decrease in signal intensity below the threshold in combined cell pools more likely.

### Cellular expression patterns reveal the road map to AM formation

**Figure 5:**
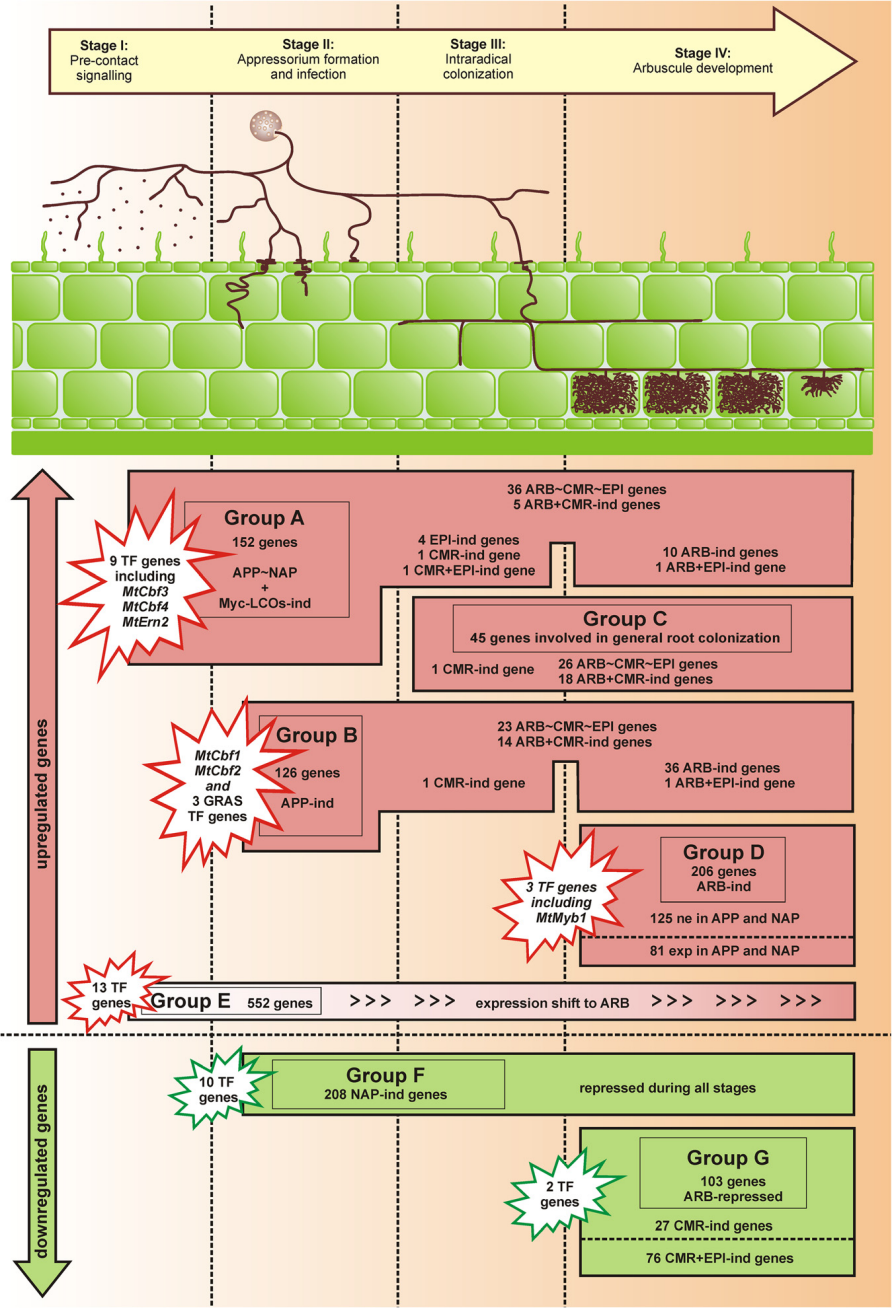
**A roadmap of cell-type specific gene expression during sequential stages of AM.** Expression patterns of 1392 *M. truncatula* genes during four AM stages. Activated genes are shown in pink, repressed genes in green. Selected transcription factor genes (Figure 6) are highlighted. Group **A**: Genes with equal expression in APP and NAP that are induced by Myc-LCOs [10], thus probably activated before contact (stage I). Genes which were also expressed in mature mycorrhizal roots are listed. Group **B**: Genes induced in APP in comparison to NAP, thus being activated during initial intracellular infection (stage II). Genes also transcribed in mature mycorrhizal roots are listed. Group **C**: Genes with equal expression in all cell-types from mature mycorrhizal roots (ARB~CMR~EPI), induced in ARB and CMR alike (ARB+CMR) or being CMR-induced. Since these were not activated by Myc-LCOs or during appressorium formation, but induced in whole mycorrhizal roots (AM core set, [32]), they are probably activated during intraradical hyphal spread (stage III) and needed for general root colonization. Group **D**: Genes preferentially expressed in ARB. Whereas transcripts of 125 genes were not found in APP or NAP, 81 genes were only weakly expressed in APP or NAP, indicating specific or predominant activation during arbuscule formation (stage IV). Group **E**: Genes induced in ARB, and equally expressed in APP and NAP, thus undergoing an expression shift towards arbusculated cells. Group **F**: Genes induced in NAP in comparison to APP, thus being repressed upon infection. Group **G**: Genes exclusively induced in CMR or in CMR as well as EPI, thus being repressed during arbuscule formation. Abbreviations: LCOs, lipo-chitooligosaccharides; APP, appressorial areas; NAP, non-appressorial areas; ARB, cortical cells containing arbuscules; CMR, cortical cells from mycorrhizal roots; EPI, epidermal cells from mycorrhizal roots; ~, equal expression; ind, induced; exp, expressed; ne, not expressed.

To draw a comprehensive picture of gene activity during AM symbioses and deduce possible gene functions, results obtained on cellular expression patterns during early infection events and mature mycorrhizal roots were combined. In Additional file 8, results are shown for all 52796 *Medicago* probe sets. The picture was completed by comparisons to gene expression patterns in whole mycorrhizal roots [32] or in seedling roots exposed to diffusible fungal signal molecules [10]. Based on these results, genes were divided into four main groups that correspond to the stages of AM symbioses defined previously [32]. Three additional groups are proposed for genes either repressed during initial infection or during arbuscule formation, and for genes which experience a shift of expression and become preferentially activated in arbusculated cells during fungal colonization. In the following, these seven groups (A-G) will be discussed with regard to their relevance during the four stages of AM development (Figure 5, Additional file 9).

#### Stage I: Pre-contact signalling via diffusible factors

The establishment of an AM symbiosis is initiated by a cross-talk between the two partners via diffusible signal molecules. Whereas strigolactones were identified on the plant side [8], mycorrhizal fungi exude lipochitoligosaccharides (LCOs, [9]), and probably additional molecules activating the first steps of the signal cascade in host plants [7]. In our analysis, this initial stage is represented by APP and NAP samples. Although the majority of genes are expressed at equal levels in APP and NAP (Additional file 8B), both cell pools were derived from root areas containing first infection sites and consequently, both were subjected to potential diffusible signal molecules. Systemic reactions triggered by the presence of the fungus are thus likely to be detected in both cell-types. This is in line with the observation that 152 genes (Group A, Additional file 9) which displayed an equal expression in APP and NAP are also induced by Myc-LCOs [10]. Genes related to secondary metabolism were most prominent among them (Additional file 10A), which may reflect the production of signal components during this stage. Only one of these genes, *MtDxs2*, has been analyzed in detail so far. It codes for a 1-deoxy-D-xylulose-5-phosphate synthase involved in the synthesis of isoprenoid-precursors [47]. The gene was shown to be involved in maintenance of arbuscule function [40], which is in line with the ARB-specific expression pattern identified in cell-types from mature mycorrhizal roots (Figure 4B). Nevertheless, the fact that a knock-down of *MtDxs2* also led to strongly reduced expression of many other AM-related genes already hinted that the protein might be required for AM development in general [40], which would demand for the early induction and continuing expression during later stages that was found by our cell-type specific expression analysis. This dual role might be explained by the production of different metabolites via the methylerythritol phosphate (MEP) pathway, acting as rather early signal molecules like e.g. strigolactone or late signal molecules like e.g. apocarotenoids [40].

Apart from *MtDxs2*, 57 genes showed a characteristic expression pattern in cell-types from mature mycorrhizal roots (Additional file 9). Of these, 11 were found to be preferentially expressed in ARB during later mycorrhizal stages, while the expression of six genes was retained to epidermal or cortical cells (Figure 5). Nevertheless, most genes were either induced in ARB+CMR alike (5 genes) or were equally expressed in ARB, CMR, and EPI (36 genes), indicating that most of them code for proteins needed for fungal colonization in general rather than arbuscule formation.

The remaining 94 genes were expressed only transiently, especially those genes related to defense mechanisms and interestingly also genes related to cell wall and membrane biogenesis (Additional file 9). We here identified seven genes coding for enzymes targeting different components of the plant cell wall, e.g. pectin, xyloglucans, and cellulose. Since cell wall degradation and modification is often discussed to promote fungal colonization, it makes sense that some of these processes are activated already during the first contact of the two symbiotic partners.

**Figure 6:**
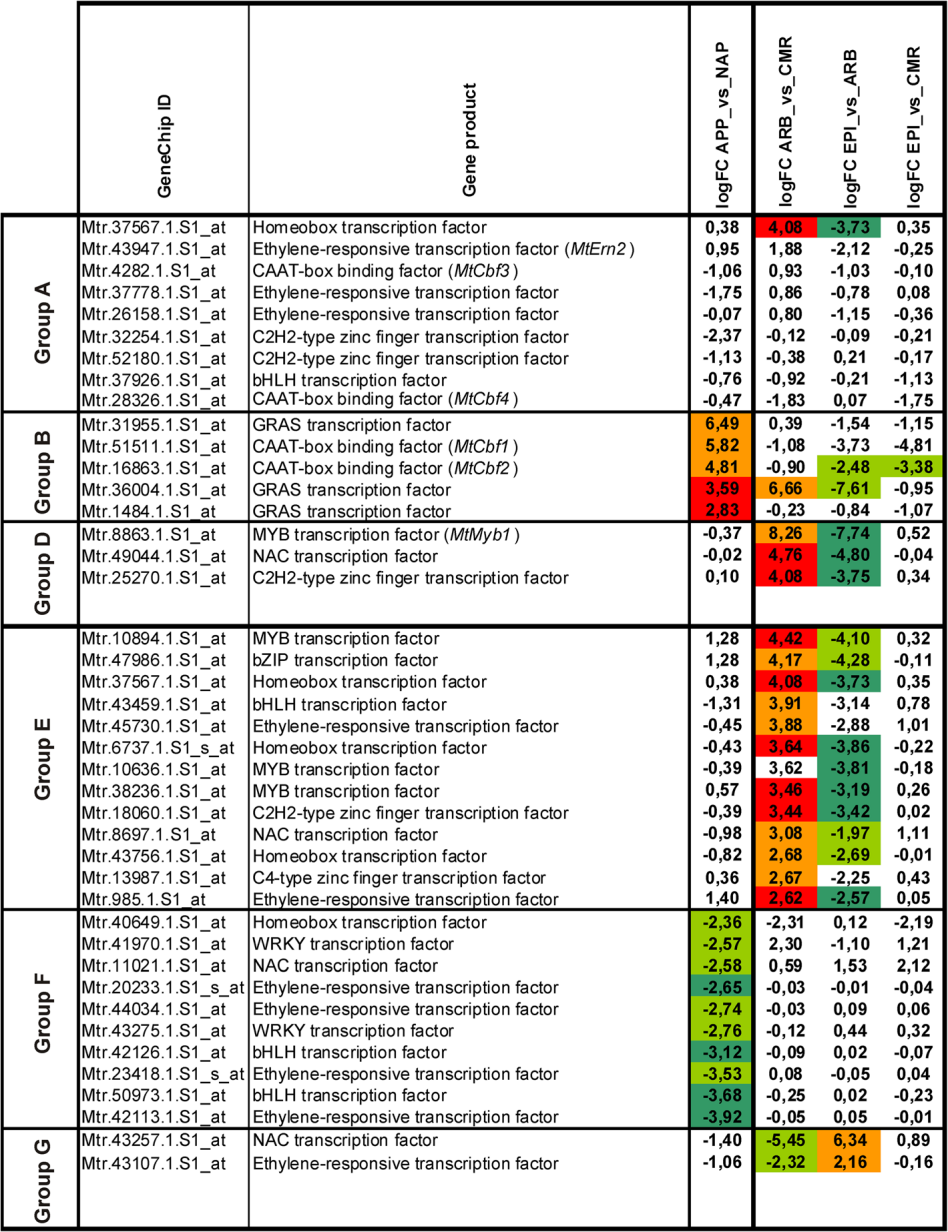
**Overview of transcription factor genes showing characteristic patterns of gene expression during AM development.** Expression pattern of 42 transcription factor genes influenced by colonization with *G. intraradices* during four distinct stages of AM development and belonging to five of the six groups of genes defined in Figure 5. *M. truncatula* genes known from previous studies are shown. Expression differences between the cell-types are indicated as defined in Figure 4. Abbreviations: ARB, cortical cells containing arbuscules; CMR, cortical cells from mycorrhizal roots; EPI, epidermal cells from mycorrhizal roots; APP, appressorial areas; NAP, non-appressorial areas; logFC, log2 fold-change.

Diffusible fungal factors are able to induce calcium spiking in *M. truncatula* roots via a signal transduction pathway common to AM and root nodule symbioses [48]. The key protein able to interpret calcium signals is a calcium-dependent protein kinase encoded by the *MtDmi3* gene [23]. Interestingly, we found five genes specifying calcium-binding proteins amongst 13 signalling-related genes (Additional file 10), which might be related to the decoding of calcium signals. While one remained expressed in all cell-types investigated during later stages, three were only active during stage I (Additional file 9). With regard to transcriptional activation downstream of calcium signalling, we identified 9 transcription factor (TF) genes (Figure 6, Additional file 11), two of them (Mtr.4282.1.S1_at. = *MtCbf3*, Mtr.28326.1.S1_at = *MtCbf4*) encoding CAAT-box TFs. These genes are of special interest, since the expression patterns identified here differ from those identified for two other CAAT-box binding TF genes, which were recently shown to be activated during fungal infection (*MtCbf1* and *MtCbf2,*[32]) and are consequently found in the group of APP-induced genes. *MtCbf3* was strongly induced by both sulphated and non-sulphated Myc-LCOs and in whole mycorrhizal roots, whereas *MtCbf4* was only slightly induced by non-sulphated Myc-LCOs and repressed in whole roots ([10,32] Additional file 11). Although the expression of *MtCbf3* was below the *MtPt4*-threshold for all cell-types from mature mycorrhizal roots, this gene was identified as equally expressed in ARB and CMR via more sensitive RT-PCR experiments [32]. This indicates that *MtCbf3* is predominantly active in the pre-contact stage, whereas transcripts accumulate at very low levels in CMR and ARB. *MtCbf4* is also active at the pre-contact stage, but does not seem to be involved in root colonization (Additional file 11). It can thus be inferred that at least *MtCbf1*, *MtCbf2*, and *MtCbf3* are activated in a sequential manner and probably control subsequent steps of the symbiosis (Figure 5). It will be interesting to investigate, whether all three gene products are essential for a successful colonization of roots by AM fungi.

#### Stage II: Initial physical contact between fungal hyphae and plant roots

In the next step of root colonization, physical contact between the two partners is established. Fungal hyphae form appressoria on the surface of the epidermis, triggering development of a pre-penetration apparatus (PPA), a structure thought to guide invading hyphae through the epidermal and the first cortical cells [24]. In our analysis, this stage is represented by genes induced in APP (126) or in NAP (208), with those induced in APP representing genes activated during the initial infection (Figure 5, Group B) and those induced in NAP representing genes repressed at this stage (Figure 5, Group F).

Among the APP-induced genes, *MtVapyrin* was also induced in roots treated with Myc-LCOs [10], together with 14 other genes. In contrast to those genes activated by Myc-LCOs prior to physical contact, APP-induced genes are probably only activated in a defined area around the site of infection and likely require a locally enhanced concentration of signal molecules in the vicinity of appressoria. Most of the APP-induced genes had a distinct expression pattern in cell-types from mature mycorrhizal roots, with 36 induced in ARB, 23 equally expressed in all cell-types, 14 induced in ARB+CMR and one gene each induced in CMR and ARB+EPI (Additional file 8B, Additional file 4). Also, 76 of these genes were already known to be induced by fungal colonization from whole root studies (Additional file 8C, Figure 5, Additional file 4), including *MtCbf1* and *MtCbf2*[32], *MtHa1*[41], *MtBcp1*[15], and *MtScp1*[12].

In contrast, the 208 NAP-induced genes displayed nearly no overlap to genes induced by fungal colonization (4 genes, Additional file 8C, Additional file 4) or Myc-LCO treatment on the level of whole roots. This is in line with the fact that most of the NAP-induced genes were either not expressed at all in cell-types from mature mycorrhizal roots (70 genes, Additional file 8B, Additional file 4) or detected as weakly expressed in one or two cell-types (86 genes, Additional file 8B, Additional file 4). Only 39 genes were equally expressed in all cell-types from mature mycorrhizal roots, while the overlap to other categories was neglectable (Additional file 8B), indicating that in most cases, transcript levels stayed low during later stages of the interaction. Interestingly, many of the NAP-induced genes also displayed a slight repression on the level of whole mycorrhizal roots (Additional file 4), with 17 of them being significantly repressed at least 2-fold by colonization with *G. intraradices*, *G. mosseae*, or both, but not by enhanced phosphate supply [32].

Together, this indicates that many of the processes relevant during later AM stages are already initiated by a first direct contact with the fungus. In combination with the distribution of functional classes (Additional file 10B), our results revealed two sets of genes either induced or repressed during this stage, consisting of genes coding for regulatory proteins as well as possible target genes. With regard to regulators, we identified 15 TFs (Figures 5 and 6, Additional file 11) and several protein kinases, as well as proteins involved in calcium-signalling. Possible targets are diverse, with genes related to the synthesis of secondary metabolites represented most strongly, in addition to genes involved in protein modification and turnover, or primary metabolism. Among the genes related to secondary metabolism, we identified a Gibberellin 2-beta-dioxygenase, which is in line with results of Ortu *et al*. [27] and Hayashi *et al*. [49], who found genes involved in the synthesis of this plant hormone to be upregulated in appressorial stages and early stages of nodulation.

#### Stage III: Intraradical growth of fungal hyphae

Beyond the initial infection site, where hyphae have crossed the epidermis and often form thick coils in the underlying cortical cells, the typical appearance of fungal structures in the cortex changes and is now dominated by thin hyphae growing in the apoplast, leading to an expansion of the infected area. It can thus be expected that genes exclusively needed during hyphal spread will be preferentially found in the group of CMR-induced genes. Nevertheless, this group was very small (32 genes, Additional file 8B, Additional file 5), and gene expression profiles in whole roots led us to the assumption that these are in fact ARB-repressed genes, which are therefore discussed in the following section. Only three genes in the CMR-induced category seem to be specifically activated during apoplastic growth of fungal hyphae in the cortex, including the gene induced by Myc-LCOs (Figure 5, Group A) and the gene induced in APP (Figure 5, Group B). Based on previous real-time RT-PCR studies, we speculated that genes exclusively related to the spread of fungal hyphae are rare [32], which is now corroborated by a genome-wide approach.

Apparently, genes expressed during stage III and IV can only be separated into those relevant for fungal colonization in general, which are also active in arbusculated cells, and genes exclusively needed during arbuscule formation, thus being preferentially transcribed in ARB. The first group will comprise genes expressed at equal levels in arbusculated and surrounding cortical cells and can thus be found within ARB+CMR-induced genes or will be expressed at equal levels throughout all cell-types colonized by AM fungi. Since both categories also contain - or even mainly consist of - genes with a constitutive expression in roots not influenced by fungal colonization, a clear correlation of gene activity with the presence of hyphae only exists for those genes identified as induced by fungal colonization on the level of whole roots (Additional file 8C). Some of these genes have already been discussed, since they are initially activated during stage I or II (Figure 5, Group A and B), but the remaining 26 genes equally expressed in ARB, CMR and EPI, as well as 18 genes induced in ARB+CMR alike (Figure 5, Group C) were neither induced by Myc-LCOs nor in appressorial areas. In both categories, the majority of genes were in addition equally expressed in APP and NAP, indicating that these genes are also transcribed in the root under non-symbiotic conditions and that their activity is only enhanced during propagation of the infected area in the root. Notably, only one TF gene was included in Group C (Figure 5), which supports the hypothesis that after a successful infection was initiated, intraradical growth of hyphae is largely independent of the activation of additional plant genes.

#### Stage IV: Arbuscule formation

The formation of arbuscules represents the final and most intimate step of the AM symbiosis. Fungal hyphae penetrate cells of the inner cortex and proliferate to highly branched structures, providing an extremely enhanced surface. Arbuscules remain surrounded by a membrane of plant origin, called the periarbuscular membrane (PAM), hosting specific transporters like the phosphate transporter MtPt4 [50] or the recently identified ABC-transporters MtStr and MtStr2 [51]. Since arbuscules are the functional units enabling the bidirectional transfer of nutrients between the symbiotic partners, many of the transcriptional changes observed in AM roots relate to the formation of those structures. This is also mirrored in our results, since the majority of genes with a cell-type specific induction were 808 ARB-induced genes (Figure 3A).

Our identification of ARB-induced genes is validated by a comparison to recent results from Gaude *et al*. [33], who compared gene expression in arbusculated and non-colonized cortical cells from *M. truncatula* AM roots to gene expression in cortical cells of non-mycorrhizal roots. A calculation of the relative induction ratios between arbusculated and cortical cells from AM roots revealed that 81 of the 100 genes we detected as most strongly induced or specifically expressed in arbusculated cells were also found to be activated by Gaude *et al*. [33] (Additional file 12). These included the AM marker genes *MtPt4*[36], *MtBcp1*[15], *MtGlp1*[39], *MtLec5*[14], *MtNip1*[52], *MtMyb1*[12], and *MtGst1*[13]. In general, the expression ratios between arbusculated and cortical cells reported by Gaude *et al*. [33] on the basis of cryo-sections were considerably lower than those specified here. As an example, an ~60-fold less induction of *MtPt4* was described [33]. These differences point to a lower degree of contamination between cell-types in our wax-embedding protocol.

Of the 808 genes classified as ARB-induced in our analysis, 125 genes were not present in APP or NAP cell-types (Additional file 8B). With 97 genes, the majority of these were ARB-specific and in addition, most of the genes were detected as induced by fungal colonization on the level of whole roots as well (Additional file 8B). This group can therefore be regarded as specifically activated during stage IV of the symbiotic interaction with a typically exclusive expression in arbusculated cells, although a few genes with additional expression in the surrounding cells were also found, e.g. the *MtMyb1* TF gene [12]. The functional classification of these 125 ARB-related genes revealed that they cover a broad spectrum of cellular processes (Additional file 10C). As could be expected, genes involved in transport and metabolism are highly represented, including the phosphate transporter gene *MtPt4*[36]. Apart from these, genes involved in arbuscule-specific signal transduction and transcriptional regulation were identified (Figure 5, Additional file 11). Also, genes encoding proteins involved in cell wall rearrangement and members of gene families known to be specifically induced in symbiotic tissues of *M. truncatula* were identified in this group, e.g. the annexin gene *MtAnn2*[38]. The most prominent functional classes were posttranslational modification and protein turnover as well as defense mechanisms. We could already show that some defensin and chitinase genes are preferentially expressed in arbusculated cells [32]. Therefore, these seem to be related to arbuscule formation or the control of arbuscule-lifespan, rather than representing typical defense responses. Induction of genes related to posttranslational protein modification and protein turnover has often been reported for mycorrhizal roots [12,15] and like the expression of defensin genes has been associated with the transient nature of single arbuscules. This demands for a tight control of fungal hyphae in these cells and break-down of fungal material, once the arbuscules start to degenerate. A similar function has been proposed for *MtTi1*, a gene encoding a trypsin inhibitor specifically expressed in arbusculated cells [37]. Our data support this hypothesis for *MtTi1* and the 18 other genes from this functional class. In addition, the 81 genes we found to be ARB-induced and only weakly expressed in APP or NAP (Additional file 8B) are probably involved in processes related to arbuscule formation and function. Together with the 125 genes discussed above, they form a large group of genes exclusively or predominantly active during stage IV of the symbiosis (Figure 5, Group D).

In addition to the specific upregulation of the genes discussed above, arbuscule formation will probably also require the cell-specific deactivation or downregulation of genes, which are otherwise expressed in the whole cortex. Such genes may be found in the category of CMR-induced genes, consisting of 32 genes with high transcript levels in CMR and significantly lower or non-detectable transcript levels in ARB and EPI (Figure 3A, Additional file 8B). Similarly, the 78 CMR+EPI-induced genes (Figure 3A, Additional file 8B) might in fact represent ARB-repressed genes, which are otherwise transcribed in the cortex and the epidermis. This is in line with the observation that nearly all genes from these two categories showed no significant expression differences between APP and NAP, indicating that they are also expressed under non-symbiotic conditions. Together they are part of the group of ARB-repressed genes (Figure 5, Group G), except for those four genes already discussed above. Interestingly, the most prominent functional class among the CMR-induced genes, which was also represented among those induced in CMR+EPI, were genes related to defense mechanisms (Additional file 10 D+E). In contrast to the defensin genes induced in arbusculated cells that were discussed above, none of these genes were induced on the level of whole roots. Besides, many of the defensin genes induced in ARB code for gamma thionins which we proposed to play a role during the typical ramification of fungal hyphae in these cells [32], whereas the ARB-repressed defense-related genes predominately encode peroxidases, representing a more general defense mechanism, which seems to be down-regulated when intracellular growth of fungal hyphae for arbuscule-formation is required. Only two of the CMR-induced genes and eleven of the CMR+EPI-induced genes were downregulated by fungal colonization on the level of whole roots, although many showed a slight non-significant reduction as observed for the NAP-induced genes, indicating that the downregulation in arbusculated cells is a transient event that might be closely correlated to the life-span of arbuscules and can thus only be detected on a cellular level.

Interestingly, a considerable overlap of 36 genes between those induced in ARB and APP was identified (Additional file 8B; Figure 5, Group B). A process relevant in both arbusculated cells and appressorial areas is the intracellular passage of fungal hyphae, whereas in the surrounding cortex the fungus mainly proceeds via the intercellular space. Genes activated in appressorial areas as well as arbusculated cells may therefore be of special importance to allow the intracellular accommodation of AM fungi. This is validated by the fact that *MtVapyrin*, which is one of the AM marker genes from this group, was shown to be relevant for initial infection as well as for arbuscule formation [42]. In addition, *MtVapyrin* is also essential for infection of root nodules by rhizobia, where an infection thread similar to the PPA is formed by the plant to guide symbiotic bacteria into the developing nodule [53]. Additional AM marker genes in this group were the H^+^ ATPase gene *MtHa1* and the *MtBcp1* gene encoding a blue copper protein. Two more genes encoding blue copper proteins were part of this group together with two genes encoding lectins and one encoding a germin-like protein (Additional file 13). This observation sheds new light on the function of these proteins, which were so far only related to arbuscule formation [14,15,39], but not to infection. Finally, 258 genes were induced in ARB and EPI cell types (Additional files 5, 8). This indicates that arbuscules as the main interface between fungus and plant have functional similarities to epidermal cells, representing the interface with the environment.

The majority of ARB-induced genes was equally expressed in APP and NAP (Additional file 8B), indicating that those are expressed in the outer cortex or in epidermal cells already at the outset of the symbiosis. Of these 562 genes, only 40 were induced by fungal colonization on the level of whole roots [32], although the majority displays a strong induction in ARB. This raises the question, whether the expression of the remaining 522 genes is related to the presence of AM fungi in the root or is influenced by other factors. It is e. g. possible that genes identified as ARB-induced may simply be more active in the inner cortical cells, irrespective of fungal colonization. Nevertheless, this explanation is not in line with the observation that 164 genes in this group were ARB-specific in mature AM roots. We therefore conclude that arbuscule formation induces a massive shift in gene expression patterns, leading to either preferential or specific accumulation of transcripts in this new cell-type, while gene activity on the whole root level largely remains the same (Figure 5, Group E). This is supported by the observation that the largest group of 13 TF genes were associated with this pattern of gene expression (Figures 5 and 6).

An example for genes included in group E is *MtSucS1*, a gene encoding a sucrose synthase providing hexoses to the symbiotic partner. This gene was expressed in arbusculated and the surrounding cortical cells, but with a strong promoter induction in the former [54] and is essential for the establishment of an effective symbiosis, since a knock-down results in early arbuscule senescence and impaired nutrient flow between plant and fungus [55]. Nevertheless, an induction of this gene was not detected on the level of whole roots (Additional file 9), since in non-mycorrhizal roots, the gene displays a strong induction in the vascular tissue and a weak, even expression throughout the cortex [54]. Due to this, genes with similar expression patterns will not be detected on the level of whole roots, but are covered by our cellular approach.

Of special interest in group E are genes related to intracellular trafficking, secretion and vesicular transport (Additional file 14), since exocytosis recently moved into focus with respect to intracellular accommodation of fungal and bacterial symbionts [56,57]. It could be shown that the secretion system of root cells is activated and concentrated in the area of fungal penetration [56]. Two *M. truncatula* genes encoding v-SNARE proteins involved in recognition and specific fusion of vesicles with target membranes are essential for arbuscule formation [57], indicating that exocytotic processes are involved in PAM formation. The genes identified here therefore represent promising candidates for further elucidation of the processes related to the cellular reprogramming required for arbuscule formation.

## Conclusions

Our study was intended to fill the gap between the identification of genes activated during AM interactions in plant roots and the time-consuming analysis of expression patterns for single genes. Via a genome-wide analysis of gene expression in different cell-types representing distinct stages of AM development, we were able to provide a substantially increased spatial and temporal resolution that allowed a true differentiation between early infection events and a functional mature mycorrhiza, leading to an identification of subsets of genes governing the sequential reprogramming of host roots towards the accommodation of fungal microsymbionts. Our analysis provided not only information on the cell-specific activity of genes known from transcriptome studies using pooled tissue samples, but moreover identified the differential expression of novel genes and revealed a fine-tuned adjustment of transcript accumulation within root tissues in response to fungal colonization. Together with other studies on cell-specific gene transcription in AM roots, the expression data reported here will provide a valuable resource to support and facilitate the functional analysis of genes mediating the sequential reprogramming of root tissues in the course of an AM symbiosis.

## Methods

### Plant growth and inoculation with arbuscular mycorrhizal fungi

*Medicago truncatula* Gaertn ‘Jemalong’ genotype A17 seeds were surface sterilized and scarified as reported by Hohnjec *et al*. [54]. Plants were grown in the climate chamber (humidity: 70%; photosynthetic photon flux: 150 μmol m^-2^ s^-1^) at a 16-h light (23°C) and 8-h dark (18°C) regime and were fertilized with half-strength Hoagland solution containing 20 μM phosphate and an additional 2mM NH_4_NO_3_. Those plants grown for the collection of root cortical cells containing arbuscules (ARB), root cortical cells from mycorrhizal roots (CMR), and root epidermal cells from mycorrhizal roots (EPI) were mycorrhized after 2 weeks with *Glomus intraradices* (recently renamed *Rhizophagus irregularis* (Błaszk., Wubet, Renker, and Buscot) C. Walker and A. Schüßler comb. nov., [34]), and mycorrhizal roots were harvested at around 21 days post inoculation (dpi) as described previously [32]. In contrast, plants grown for the collection of appressorial root areas (APP) and the corresponding non-appressorial controls (NAP) were mycorrhized after 3 weeks and roots were harvested at 5-6 dpi. This procedure resulted in a larger root system at the time of mycorrhization and an enhanced number of infection units. Since no infection sites were visible at 4 dpi, all appressorial areas had a maximum age of 48 hours.

### Tissue embedding, tissue sectioning, and laser-microdissection

Roots were embedded using the Steedman’s wax protocol [31] with the modifications reported in [32]. For the collection of ARB, CMR, and EPI cell pools, the whole root system of mycorrhizal plants was cut into approximately 1 cm pieces and embedded. For the collection of APP and NAP cell pools, an additional screening step was included. The root system of the mycorrhizal plant was submerged in ink staining solution [58] prepared with RNAse free water and 8% (v/v) glacial acetic acid for 5-10 min on ice. Roots were then transferred into 0.8% (v/v) acetic acid solution prepared with glacial acetic acid and RNAse free water and screened for extraradical hyphae using a stereo microscope. Root segments with extraradical hyphae were embedded for APP samples, while distant root segments with no visible fungal structures were embedded for NAP samples. Longitudinal sections of 12 μm on glass slides were obtained as described previously [32]. Also, the P.A.L.M. microbeam system with a Capmover (Zeiss, München, Germany) was used for laser-microdissection and pressure catapulting as described before [32]. For each cell-type, three biological replicates were produced, based on distinct rounds of plant cultivation and root embedding. For ARB, CMR, and EPI cells, each biological replicate consisted of three technical replicates with approximately 1000 cells each, which were pooled after RNA isolation and amplification. For NAP and APP cells, biological replicates consisted of one technical replicate of approximately 100 appressorial or control areas. These on average comprised 10 cells, leading to a final number of 1000 cells in each sample.

### RNA isolation and amplification

Total RNA was isolated from laser-microdissected cells using the RNeasy Micro kit (Qiagen, Hilden, Germany). 350 μl of RLT buffer containing β-mercaptoethanol were added to each sample followed by a 30-min incubation at room temperature. The lysate was spun down for 5 min at 13400 g, mixed 1:1 with ethanol absolute, and transferred to the clean-up column. On-column DNAse I digestion was performed according to the manufacturer’s instructions. RNA was amplified using the TargetAmp 2-round Biotin aRNA amplification kit (Epicentre Biotechnologies, Madison, USA). For each sample, several rounds of amplification were carried out and pooled subsequently. Quantity and quality of total RNA as well as T7-amplified biotinylated aRNA was checked via capillary electrophoresis in RNA 6000 pico and nano assays, respectively, using an Agilent 2100 bioanalyzer (Agilent Technologies, Böblingen, Germany). Additionally, the pooled samples of T7-amplified biotinylated aRNA were checked via real-time RT-PCR for the presence or absence of selected marker genes. Primer design and real-time RT-PCR conditions were already described in [32].

### *Medicago* GeneChip hybridizations

Biotinylated aRNA obtained for each sample was fragmented as recommended (GeneChip 3′IVT express kit, Affymetrix, Santa Clara, California, USA). The size distribution of the fragmented aRNA was assessed via an Agilent bioanalyzer (Agilent Technologies, Böblingen, Germany) using an RNA 6000 assay. The fragmented aRNA was added to a 300 μl hybridization cocktail also containing hybridization controls. 200 μl of the mixture were hybridized to GeneChips for 16 h at 45°C. Standard post-hybridization wash and double-stain protocols (FS450_0001; GeneChip HWS hit; Affymetrix, Santa Clara, California, USA) were used on an Affymetrix GeneChip fluidics station 450. GeneChips were scanned on an Affymetrix GeneChip scanner 3000 7G.

### Evaluation of data from *Medicago* GeneChip hybridizations

Cel files obtained from *Medicago* GeneChip hybridizations were analysed using Robin [59]. Normalization was performed via the Robust Multichip Average algorithm across mature mycorrhizal stages (ARB, CMR, EPI) and early infection events (APP, NAP). Intensity values calculated for each probe set were log2-transformed and averaged across all three biological replicates. Log2 differences were evaluated statistically via Student’s t-tests implemented in the Robin software [59]. Since *Medicago* GeneChips are based on gene models from EST and genomic sequences, the number of probe sets somewhat exceeds the number of genes represented. Nevertheless, we refer to genes instead of probe sets in this work for reasons of simplicity. Original annotations of genes represented by *Medicago* GeneChips were updated via the SAMS software [60] that incorporates annotations from the *Medicago* genome version 3.5 [45]. The functional classification of genes was based on automated KOG categories or on manually assigned SAMS annotations, in case no KOGs were found. Venn diagrams were drawn using the VENNY software [61].

### Availability of supporting data

The data sets supporting the results of this article are available in the Gene Expression Omnibus repository, http://www.ncbi.nlm.nih.gov/gds?term=GSE42748.

## Competing interests

The authors declare that they have no competing interests.

## Authors’ contributions

CH carried out the experimental work and drafted the manuscript. HK conceived the experiments, participated in data evaluation, and helped to draft the manuscript. All authors read and approved the final manuscript.

## Supplementary Material

Additional file 1 Complete gene expression dataset obtained from three cell-types of mature mycorrhizal roots (ARB, CMR, EPI). Click here for file

Additional file 2 Complete gene expression dataset obtained from two cell-types of roots harbouring first infection units (APP, NAP). Click here for file

Additional file 3 Genes detected as expressed in the three cell-types from mature mycorrhizal roots (ARB, CMR, EPI). Click here for file

Additional file 4 Types of gene expression in the APP and NAP samples. KOG classifications and comparisons to the AM core set are included. Click here for file

Additional file 5 **Types of gene expression in the ARB, CMR, and EPI samples.** KOG classifications and comparisons to the AM core set are included. Click here for file

Additional file 6 Overview of fungal genes detected in this study. Click here for file

Additional file 7 **Functional classification of fungal genes expressed in appressorial cell pools.** Genes were grouped according to their KOG classification or SAMS [60] annotation, in case no KOG class was available. The 100 genes classified as “Unknown function” are not included. Click here for file

Additional file 8 **Comparison of gene expression in cell-types from mature mycorrhizal stages (ARB, CMR, EPI) and early infection events (APP, NAP).** Results for all 52796 *Medicago* probe sets are shown. The expression categories for mature mycorrhizal stages (ARB, CMR, EPI) are shown in columns, those of for early infection events (APP, NAP) in rows. Additionally, genes that were detected as expressed according to the *MtPt4* expression threshold, but did not show significant expression differences between cell-types (columns “ARB/CMR/EPI-exp” and “APP/NAP-exp”), as well as genes that were not expressed, are listed for both datasets (columns “ne in ARB/CMR/EPI” and “ne in APP/NAP”). The numbers of genes in each combination of categories is given. In the last column and the last row, results are summed up. In **A**, 217 fungal genes identified in Additional file 6 are included, whereas these were kept out in **B**, reducing the total number to 52579. **C** shows, how many of the genes in each comparison were already known to be significantly activated in whole roots at least 2-fold after colonization with *Glomus intraradices*, *Glomus mossae*, or both; but not being induced by enhanced phosphate supply [32]. As an example, 35 of the 36 ARB- and APP-induced *Medicago truncatula* genes were also identified as induced by fungal colonization on the level of whole roots, whereas one gene was not. Abbreviations: ARB, cortical cells containing arbuscules; CMR, cortical cells from mycorrhizal roots; EPI, epidermal cells from mycorrhizal roots; APP, appressorial areas; NAP, non-appressorial areas; ind, induced; ~, equal expression; exp, expressed; ne, not expressed; * genes were expressed in one or two of the three cell-types, but with no significant induction in comparison to the others; # genes were expressed in one of the two cell-types, but with no significant induction in comparison to the other. Click here for file

Additional file 9 **List of seven groups of genes as defined in Figure **5. Click here for file

Additional file 10 **Functional classification of genes from selected expression categories.** Genes were grouped into functional classes according to their automated KOG classification or their SAMS [60] annotation, in case no KOG classes were available. Where possible, members of AM-related gene families (annexins, blue copper proteins, germin-like proteins, lectins) were grouped separately. Genes grouped into the “Unknown function” category (51 in A, 164 in B, 47 in C, 16 in D, 44 in E) are not included. Black bars indicate the proportion of genes significantly induced (or repressed in the case of NAP-induced genes on the left side of panel B) in whole roots at least 2-fold by colonization with *G. intraradices*, *G. mossae*, or both; but not by enhanced phosphate supply [32]. Striped bars refer to genes which were not significantly induced or repressed under those conditions. **A:** Genes displaying no significant expression differences between APP and NAP and being induced at least 1.5 fold (p≤0.05) in roots treated with Myc-LCOs [10]. **B:** Genes induced in NAP or APP areas (logFC≥2.32; p≤0.05). APP-induced genes are depicted on the right side, NAP-induced (or APP-repressed) genes on the left. **C:** Genes induced in ARB (logFC≥1.32; p≤0.01) that were not expressed in APP and NAP areas. **D:** Genes induced in the CMR cell-type (logFC≥1.32; p≤0.01). **E:** Genes induced in CMR and EPI cell-types (logFC≥1.32; p≤0.01). Abbreviations: Sec., Secondary; Posttransl., Posttranslational. Click here for file

Additional file 11 Detailed overview of selected transcription factor genes including expression data from mature mycorrhizal roots [32] and pre-contact stages [10]. Click here for file

Additional file 12 **Comparison of the 100 genes most strongly induced in ARB to the expression data obtained by Gaude***et al*. [33]. Click here for file

Additional file 13 **Functional classification of genes induced in ARB and APP cell-types.** Genes were grouped according to their KOG classification or SAMS [60] annotation, in case no KOG class was available. Black bars refer to genes which were also significantly induced in whole roots at least 2-fold by colonization with *G. intraradices*, *G. mossae*, or both; but not by enhanced phosphate supply [32]. Striped bars refer to genes not induced under these conditions. AM-related gene families (annexins, blue copper proteins, germin-like proteins, lectins) were grouped separately. The 17 genes classified as “Unknown function” are not included. Click here for file

Additional file 14 **Functional classification of genes induced in ARB and equally expressed in APP and NAP.** Genes were grouped according to their KOG classification or SAMS [60] annotation, in case no KOG class was available. Black bars refer to genes which were also significantly induced in whole roots at least 2-fold by colonization with *G. intraradices*, *G. mossae*, or both; but not by enhanced phosphate supply [32]. Striped bars refer to genes which were not induced under these conditions. AM-related gene families (annexins, blue copper proteins, germin-like proteins, lectins) were grouped separately. The 211 genes classified as “Unknown function” are not included. Click here for file
