# Supplementary figures and images for: A roadmap of cell-type specific gene expression during sequential stages of the arbuscular mycorrhiza symbiosis
Source: BMC Genomics. 2013 May 7;14:306. doi: 10.1186/1471-2164-14-306 (PMC3667144; doi:10.1186/1471-2164-14-306)

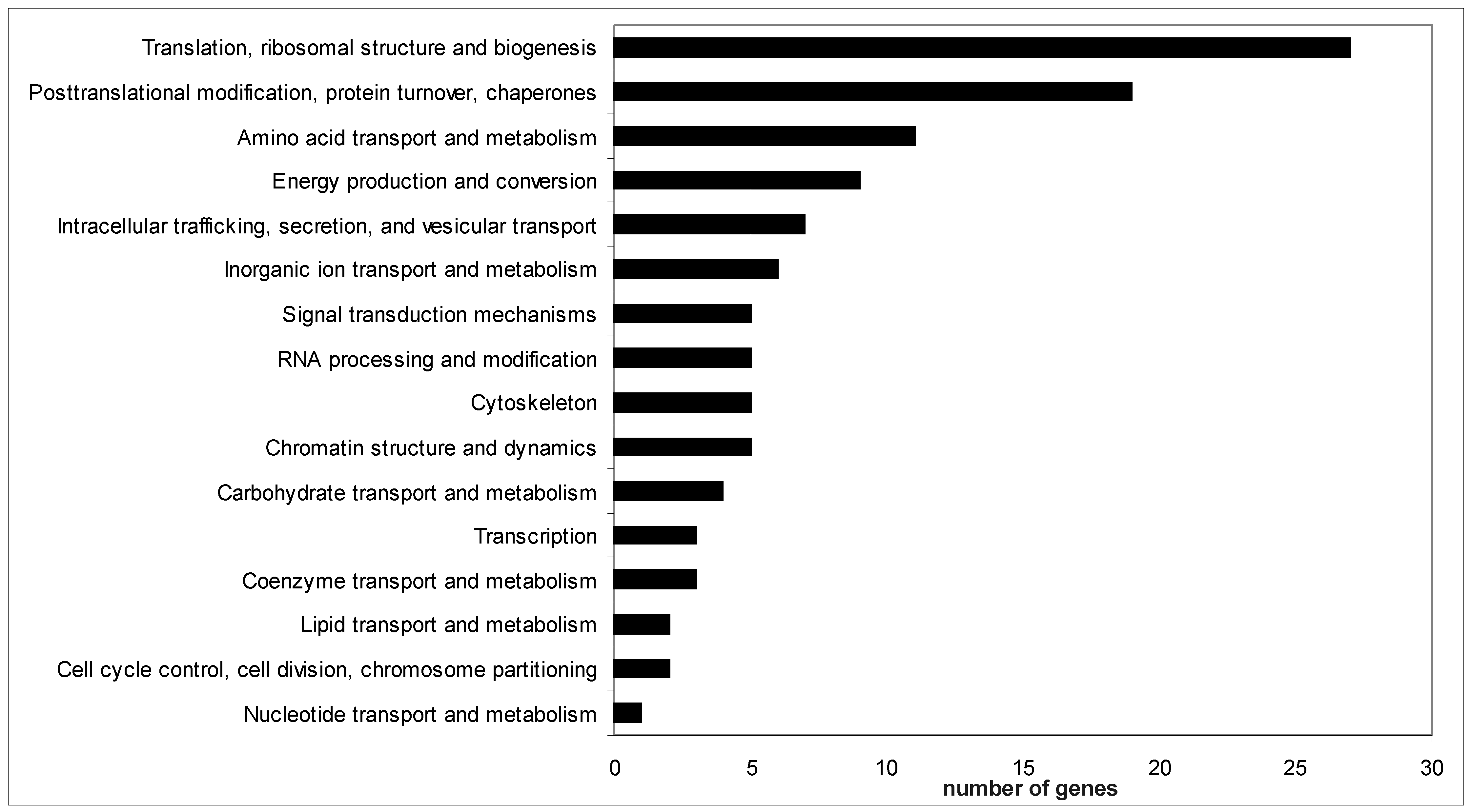

Supplement: Additional file 7 — Functional classification of fungal genes expressed in appressorial cell pools. Genes were grouped according to their KOG classification or SAMS [60] annotation, in case no KOG class was available. The 100 genes classified as “Unknown function” are not included. [file 1471-2164-14-306-S7.tiff]

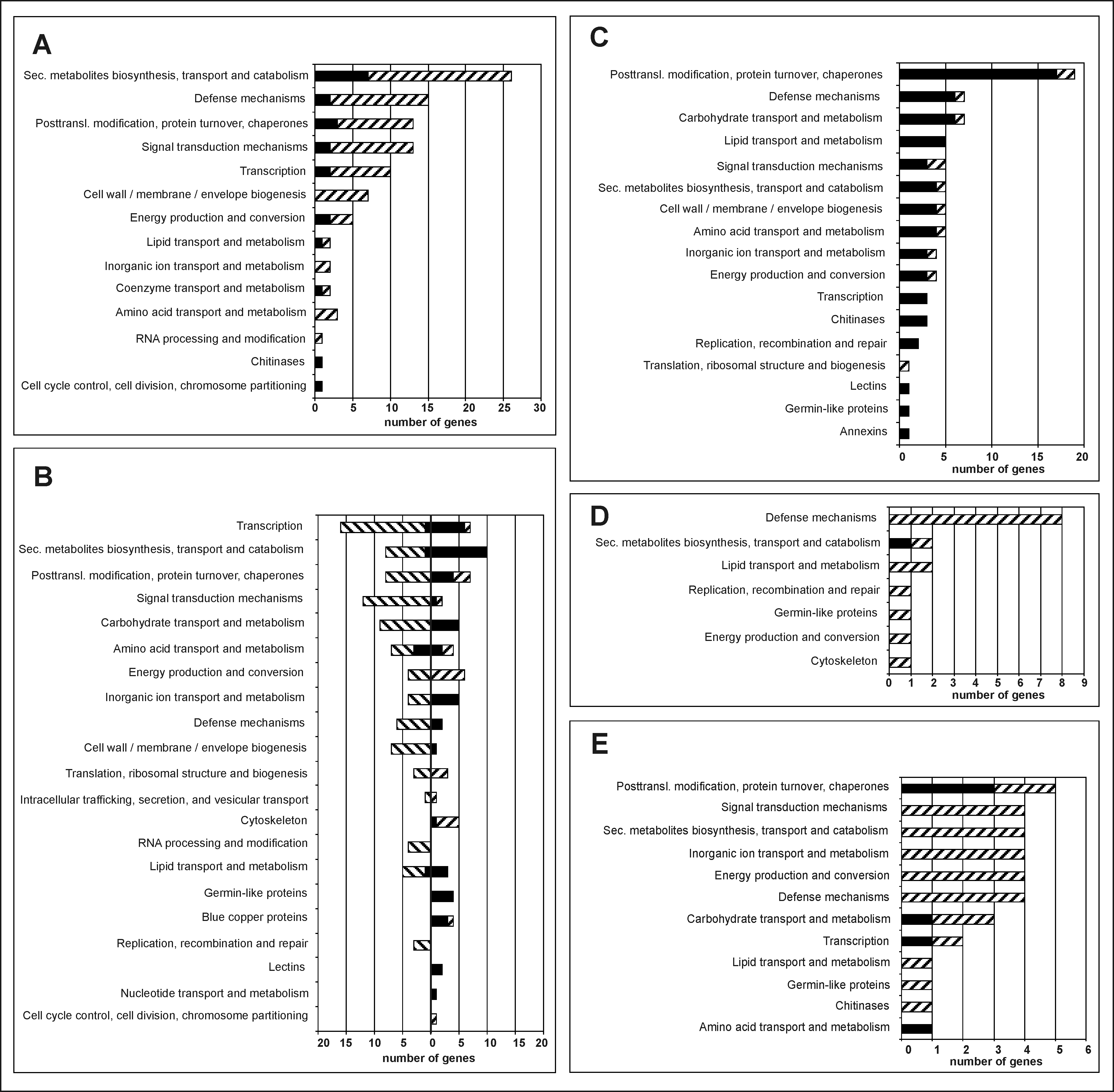

Supplement: Additional file 10 — Functional classification of genes from selected expression categories. Genes were grouped into functional classes according to their automated KOG classification or their SAMS [60] annotation, in case no KOG classes were available. Where possible, members of AM-related gene families (annexins, blue copper proteins, germin-like proteins, lectins) were grouped separately. Genes grouped into the “Unknown function” category (51 in A, 164 in B, 47 in C, 16 in D, 44 in E) are not included. Black bars indicate the proportion of genes significantly induced (or repressed in the case of NAP-induced genes on the left side of panel B) in whole roots at least 2-fold by colonization with G. intraradices, G. mossae, or both; but not by enhanced phosphate supply [32]. Striped bars refer to genes which were not significantly induced or repressed under those conditions. A: Genes displaying no significant expression differences between APP and NAP and being induced at least 1.5 fold (p≤0.05) in roots treated with Myc-LCOs [10]. B: Genes induced in NAP or APP areas (logFC≥2.32; p≤0.05). APP-induced genes are depicted on the right side, NAP-induced (or APP-repressed) genes on the left. C: Genes induced in ARB (logFC≥1.32; p≤0.01) that were not expressed in APP and NAP areas. D: Genes induced in the CMR cell-type (logFC≥1.32; p≤0.01). E: Genes induced in CMR and EPI cell-types (logFC≥1.32; p≤0.01). Abbreviations: Sec., Secondary; Posttransl., Posttranslational. [file 1471-2164-14-306-S10.tiff]

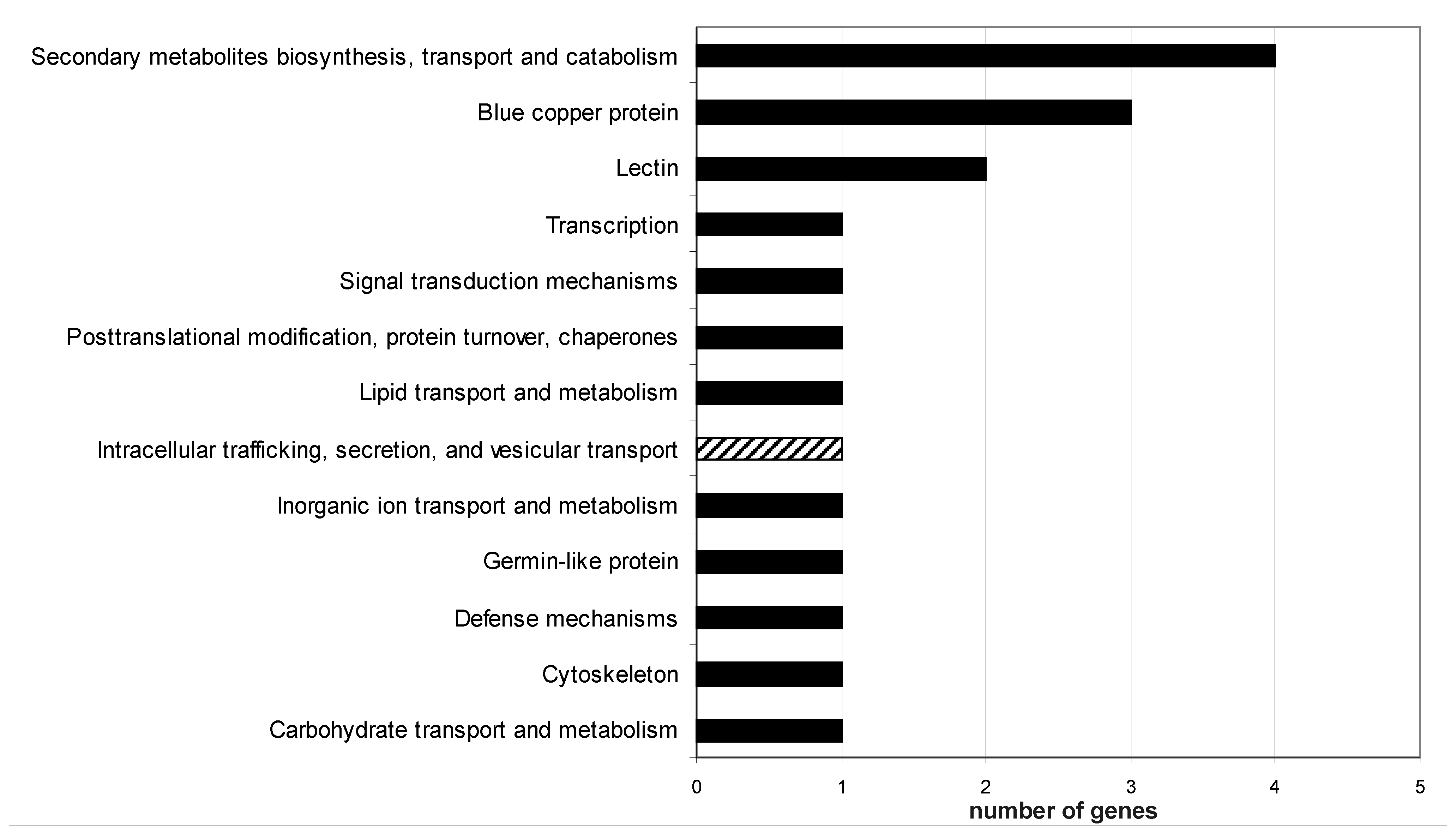

Supplement: Additional file 13 — Functional classification of genes induced in ARB and APP cell-types. Genes were grouped according to their KOG classification or SAMS [60] annotation, in case no KOG class was available. Black bars refer to genes which were also significantly induced in whole roots at least 2-fold by colonization with G. intraradices, G. mossae, or both; but not by enhanced phosphate supply [32]. Striped bars refer to genes not induced under these conditions. AM-related gene families (annexins, blue copper proteins, germin-like proteins, lectins) were grouped separately. The 17 genes classified as “Unknown function” are not included. [file 1471-2164-14-306-S13.tiff]

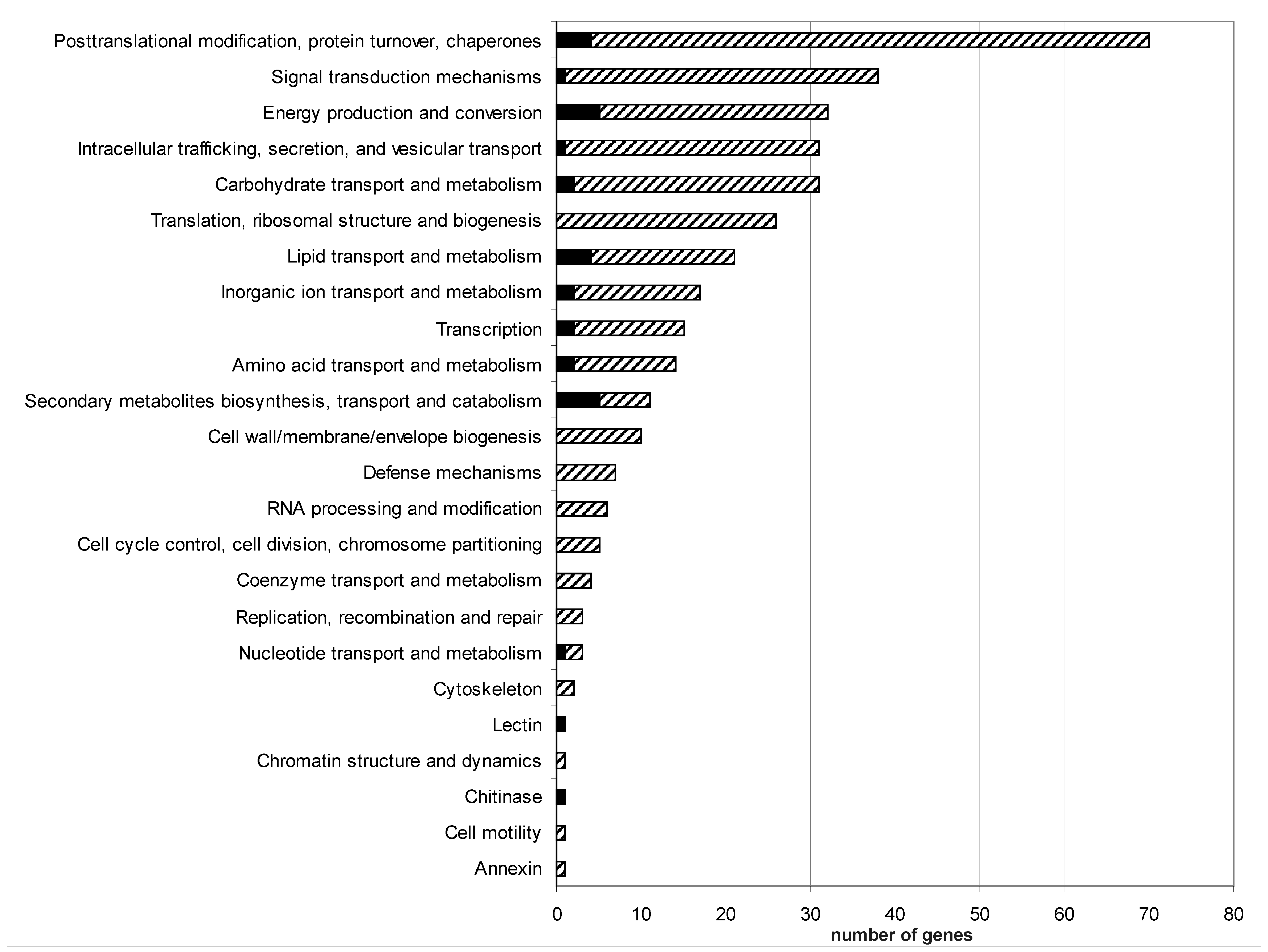

Supplement: Additional file 14 — Functional classification of genes induced in ARB and equally expressed in APP and NAP. Genes were grouped according to their KOG classification or SAMS [60] annotation, in case no KOG class was available. Black bars refer to genes which were also significantly induced in whole roots at least 2-fold by colonization with G. intraradices, G. mossae, or both; but not by enhanced phosphate supply [32]. Striped bars refer to genes which were not induced under these conditions. AM-related gene families (annexins, blue copper proteins, germin-like proteins, lectins) were grouped separately. The 211 genes classified as “Unknown function” are not included. [file 1471-2164-14-306-S14.tiff]
